# Differential expression of centrosome regulators in Her2+ breast cancer cells versus non-tumorigenic MCF10A cells

**DOI:** 10.1186/1747-1028-9-3

**Published:** 2014-09-25

**Authors:** Mi-Young Lee, Mihaela Marina, Jamie L King, Harold I Saavedra

**Affiliations:** 1 Department of Radiation Oncology, Winship Cancer Institute, Emory University School of Medicine, C3084, 1365C Clifton Road NE, Atlanta, GA 30322, USA; 2 Cancer Biology Graduate Program, Emory University School of Medicine, 1365C Clifton Road NE, Atlanta, GA 30322, USA

**Keywords:** Centrosome amplification, Cell cycle, Her2+ breast cancer, SFRP1, MDM2, SGOL1, TTK

## Abstract

Centrosome amplification (CA) amongst particular breast cancer subtypes (Her2+ subtype) is associated with genomic instability and aggressive tumor phenotypes. However, changes in signaling pathways associated with centrosome biology have not been fully explored in subtype specific models. Novel centrosome regulatory genes that are selectively altered in Her2+ breast cancer cells are of interest in discerning why CA is more prevalent in this subtype. To determine centrosome/cell cycle genes that are altered in Her2+ cells that display CA (HCC1954) versus non-tumorigenic cells (MCF10A), we carried out a gene microarray. Expression differences were validated by real-time PCR and Western blotting. After the microarray validation, we pursued a panel of upregulated and downregulated genes based on novelty/relevance to centrosome duplication. Functional experiments measuring CA and BrdU incorporation were completed after genetic manipulation of targets (TTK, SGOL1, MDM2 and SFRP1). Amongst genes that were downregulated in HCC1954 cells, knockdown of MDM2 and SFRP1 in MCF10A cells did not consistently induce CA or impaired BrdU incorporation. Conversely, amongst upregulated genes in HCC1954 cells, knockdown of SGOL1 and TTK decreased CA in breast cancer cells, while BrdU incorporation was only altered by SGOL1 knockdown. We also explored the Kaplan Meier Plot resource and noted that MDM2 and SFRP1 are positively associated with relapse free survival in all breast cancer subtypes, while TTK is negatively correlated with overall survival of Luminal A patients. Based on this functional screen, we conclude that SGOL1 and TTK are important modulators of centrosome function in a breast cancer specific model.

## Introduction

Breast cancer is the most common form of cancer detected globally and the second leading cause of cancer-related deaths in women. Fatality from breast cancer is mostly due to metastasis and the risk of metastasis and relapse partially correlates with tumor heterogeneity. Chromosome instability (CIN) -defined as the active gain or loss of whole or fragments of chromosomes during cell division- and aneuploidy -the state of having abnormal chromosome numbers- remain uncontested sources of genetic heterogeneity and are associated with the most aggressive breast tumor types [1]. Equal segregation of chromosomes into daughter cells and the maintenance of euploidy are ensured by the two mitotic centrosomes that direct the formation of a bipolar spindle [2].

Various molecular alterations, such as expression of oncogenes and deregulation of proteins that control the centrosome cycle, the spindle assembly checkpoint or the cell cycle culminate in centrosome amplification (CA), defined as the presence of more than a pair of centrosomes within a cell [2,3]. For example, we reported that a subset of Her2+ cell lines display CA, which strongly correlated with increased protein expression of the centrosome kinases Nek2 and Plk4 [4]. Both of these proteins, when overexpressed, induced CA in mammary epithelial cells previously devoid of CA [5-7]. Emerging evidence suggests that CIN and aneuploidy, including single chromosome losses and polyploidy, can ensue from CA and/or cytokinesis defects [2,8-10]. CA has been detected in pre-malignant lesions, DCIS (ductal carcinoma in situ) and in invasive breast tumors [11-14]. In addition, CA significantly correlates with amplification of the Her2 receptor, which affects over 20% breast cancer patients, specifically patients displaying the Her2 + ER-PR- and luminal B subtypes [11,12]. Moreover, genomically unstable and more aggressive aneuploid breast cancers have a greater extent of CA and abnormal mitotic spindles compared to genomically stable aneuploid and diploid tumors. In conclusion, preventing the acquisition of CA, by targeting the proteins that enable it, could potentially prevent cancer initiation and tumor progression.

Our screening of breast cancer cell lines revealed that CA is prevalent in a subset of Her2+ cells. Therefore, we chose to use these cell lines as our model of breast cancer displaying CA. We found that E2F activators, Cdk4 and Nek2 kinases are overexpressed and/or deregulated in these cell lines compared to non-tumorigenic mammary epithelial cells [15,16]. Using genetic manipulation approaches, we identified that these proteins are required for the maintenance of CA and binucleation in Her2+ breast cancer cells. However, since these molecules regulate a plethora of biological processes, including cell cycle progression, mitosis and cell proliferation, their potential value as selective modifiers of CA is limited.

Here, we present data from gene array experiments performed in order to identify novel centrosome and cell cycle genes that might be selectively modified in Her2+ breast cancer cells versus non-tumorigenic cells. Our aim was to identify differences in gene expression between breast cancer cells with high percentages of CA and non-transformed cells. We hypothesized that genes that are selectively expressed translate into deregulated centrosome functions and might provide therapeutic targets to specifically address CA.

We identified several molecules that might establish a CA phenotype in Her2+ breast cancer cells. Specifically, breast cancer cells overexpressed GINS2, TTK, CEP192, and shugoshin 1 and have lower levels of C-Nap1, semaphorin 6A, MDM2 and SFRP1. Some of these proteins have been previously linked to CA and breast cancer progression, while others have relatively unknown functions in this context. Our findings provide relevant insight into genetic and functional differences in centrosome regulators between breast cancer and non-tumorigenic cells. Potentially, selectively expressed proteins may represent biomarkers for the identification of Her2+ tumors prone to develop CA and other forms of genomic instability, as well as novel therapeutic targets against CA to prevent tumor initiation and disease progression.

## Materials and methods

### Cell culture

All cell lines were obtained from the ATCC or from collaborators. Culture conditions for MCF10A, HCC1954, SKBR3 and JIMT-1 cells have been described [17,18].

### Data acquisition and processing

Total RNA was isolated using RNeasy mini kit (Qiagen) and subjected to quality control assessment. Affymetrix Gene Expression microarrays (Affymetrix) were used according to the manufacturer’s protocol. Raw intensities of the arrays (2 for MCF10A/pLKO.1 and 2 for HCC1954/pLKO.1 cells) were normalized using quantile normalization and then log2 transformed in the Affymetrix Human U133 platform prior to doing the analyses. Distribution of the samples was calculated and 20% top differentially expressed genes were selected for comparative analysis. Averaged data were then uploaded into Metacore where gene expression probe names were identified and differentially expressed genes (fold threshold ≥1.5) for centrosome and cell cycle GO processes were displayed and further analyzed.

### RNA extraction and real-time PCR analysis

Two μg of RNA were used to synthesize cDNA (Promega) following the manufacturer’s protocols. Next, two μL of 1:10 diluted cDNA were used for real-time PCR with iQ SYBR Green supermix (170-8880, Bio-Rad). Actin was loaded as an internal control. Primer sequences (Integrated DNA Technologies) are presented in Table 1.

**Table 1:** Primers sequences used for real-time PCR and siRNA duplexes

**Primers used for real-time PCR**	
**Genes**	**Primer sequences**
AURAK_F	5′-ATA TCT CAg Tgg Cgg ACg Ag-3′
AURAK_R	5′-TCA AAT ATC CCC gCA CTC Tgg-3′
CDC14B_F	5′-CTC CAT gAA gCg gAA AAg Cg-3′
CDC14B_F	5′-gCA AAA CAA Agg CgA TCg gT-3′
CDK1_F	5′-AAA CTg gCT gAT TTT ggC CT-3′
CDK1_R	5′-ggA gTg CCC AAA gCT CTg AA-3′
CEP192_F	5′-CCC gAg CAC TTg ATT CTg gT-3′
CEP192_R	5′-CCA CTC CAC ggg AAC ATT gA-3′
CETN2_F	5′-AgC ggA CTC CTT Tgg CTA Tg-3′
CETN2_R	5′-gCT CAg gCT TAg ggC TCA TT-3′
GINS2_F	5′-gCC gAg AAg gAg CTg gTT AC-3′
GINS2_R	5′-AAC CAg ggT TAA AAg gCC CC-3′
ROCK2_F	5′-CCC ATC AAC gTg gAg AgC TT-3′
ROCK2_R	5′-TgC CTT gTg ACg AAC CAA CT-3′
SASS6-F	5′-TAC ggA ATg AAT ggg CgT CA-3′
SASS6_R	5′-CTg TgC CTg CAA ggC TTT TT-3′
SPICE_F	5′-ggT CCC CgA gTT ggT gTA Ag-3′
SPICE_R	5′-gCg TAC CAg ATC TTC ggg Ag-3′
SGOL1_F	5′-Agg CAA AAg ATg gCC AAg gA-3′
SGOL1_R	5′-AAA gAC CTg CgT TTg CCA AT-3′
CDK14 F	5′-AAT gAg gAC ACA Tgg CCT gg-3′
CDK14_R	5′-CTg TgC CgA CAg TCT gTT CT-3′
c-Nap_F	5′-AAC CAg CTC Cgg gAg AAA Tg-3′
c-Nap_R	5′-TCT ggC ATA ggg CAC TCT CT-3′
MDM2_F	5′-CCA TgC CTg CCC ACT TTA gA-3′
MDM2_R	5′-CAg gCT gCC ATg TgA CCT AA-3′
PlexinA2_F	5′-ATT TTT CAg CCg AgA ggg Cg-3′
PlexinA2_R	5′-TTT TTC CAg CgC gAC TTT CC-3′
SEMA6A_F	5′-TgA TgC CAA ACA TgC CAA Cg-3′
SEMA6A_R	5′-gCg TCA ATg gCA Agg AAg TC-3′
SFRP1_F	5′-CTC AAC AAg AAC TgC CAC gC-3′
SFRP1_R	5′-CTC gTT gTC ACA ggg Agg AC-3′
Actin_F	5′-CgA ggC CCA gAg CAA gAg-3′
Actin_R	5′-CgT CCC AgT Tgg TAA CAA TgC-3′
TTK_F	5′-CgC AgC TTT CTg TAg AAA TggA-3′
TTK_R	5′-gAg CAT CACTTAGCGGAACAC-3′
**siRNA duplexes**	
MDM2_1 Sense	5′-rGrGrA rCrCrU rUrGrU rArCrA rArGrA rGrCrU rUrCrA rGrGA A-3′
MDM2_1 Anti-Sense	5′-rUrUrC rCrUrG rArArG rCrUrC rUrUrG rUrArC rArArG rGrUrC rCrUrU-3′
MDM2_2 Sense	5′-rCrCrA rArGrA rCrArA rArGrA rArGrA rGrArG rUrGrU rGrGA A-3′
MDM2_2 Anti-Sense	5′-rUrUrC rCrArC rArCrU rCrUrC rUrUrC rUrUrU rGrUrC rUrUrG rGrGrU-3′
SFRP1_2 Sense	5′-rGrArA rGrCrA rArCrA rGrCrU rUrCrA rGrArA rArGrA rGrCT C-3′
SFRP1_2 Anti-Sense	5′-rGrArG rCrUrC rUrUrU rCrUrG rArArG rCrUrG rUrUrG rCrUrU rCrCrU-3′
SFRP1_3 Sense	5′-rGrArA rArCrA rUrUrU rCrCrU rUrUrG rArArC rUrUrG rArUT G-3′
SFRP1_3 Anti-Sense	5′-rCrArA rUrCrA rArGrU rUrCrA rArArG rGrArA rArUrG rUrUrU rCrUrU-3′
TTK_1 Sense	5′-rGrGrArGrGrUrUrCrArArGrCrArArGrGrUrArUrUrUrCrAGG-3′
TTK_1 Anti-Sense	5′rCrCrUrGrArArArUrArCrCrUrUrGrCrUrUrGrArArCrCrUrCrCrArC-3′
TTK_2 Sense	5′-rCrCrArGrArArUrCrCrUrGrCrUrGrCrArUrCrUrUrCrArAAT-3′
TTK_2 Anti-Sense	5′-rArUrUrUrGrArArGrArUrGrCrArGrCrArGrGrArUrUrCrUrGrGrUrU-3′
SGOL1_1 Sense	5′-rGrArArArUrArUrGrUrUrCrCrUrCrUrGrGrArArUrGrGrACC-3′
SGOL1_1 Anti-Sense	5′-rGrGrUrCrCrArUrUrCrCrArGrArGrGrArArCrArUrArUrUrUrCrCrU-3′
SGOL1_2 Sense	5′-rGrGrArCrUrArCrArGrGrCrArUrGrUrGrCrCrArCrUrArCGC-3′
SGOL1_2 Anti-Sense	5′-rGrCrGrUrArGrUrGrGrCrArCrArUrGrCrCrUrGrUrArGrUrCrCrCrA-3′

### siRNA transfection

Cells were seeded overnight in either 60 mm culture dishes or in four well chamber slides (Thermo Scientific). Lipofectamine RNAiMAX (13778075, Life Technologies), along with two hundred pmoles of each of the MDM2, SFRP1, SGOL1 and TTK siRNA constructs (Integrated DNA Technologies) or 5 μL of silencer negative control siRNA #1 (50 μM) (AM4611, Life Technologies) were transfected for 48 hours. siRNA sequences are presented in Table 1.

### Immunofluorescence for Bromodeoxyuridine (BrdU) incorporation and centrosome amplification

BrdU staining was performed as described in our publication [8]. Briefly, forty-eight hours post transfection, BrdU was incubated in the media of cells grown in four well chamber slides at a final concentration of 10 μM for 30 min prior to fixing the cells in 4% paraformaldehyde for 10 min. DNA was denatured in 2 N HCl for 20 min at room temperature, followed by neutralization in 0.1 M sodium borate pH 8.5 for 2 minutes. Next, cells were permeabilized in 0.1% NP-40 solution for 10 min after three consecutive washes with PBS and then blocked in 10% normal goat serum (Life Technologies) for 1 h before incubation with anti-BrdU antibody (NA61, Calbiochem) at 4°C overnight. DAPI (1 mg/mL) counterstain was used. Five hundred cells were counted and the percentages of BrdU + cells were calculated using fluorescent microscopy.

Centrosome amplification in transiently transfected cells was addressed using four well slides. Forty-eight hours post transfection, cells were fixed in 4% paraformaldehyde for 10 min, washed in PBS, permeabilized in 0.1% NP-40 solution for 10 min and blocked in 10% normal goat serum (Life Technologies) for 1 h, followed by overnight incubation with primary antibody against pericentrin (ab4448, Abcam). Alexa Fluor-conjugated antibodies (A11008, A11001 or A21069, Life Technologies) were used as secondary antibodies by incubating 1 h at room temperature. As a counter staining, DAPI (1 mg/mL) was applied. Two hundred cells were counted and cells with ≥3 pericentrin positive cells were presented as percentages.

### Western blotting

Western blotting was performed according to our published protocols [8,15,19]. The following primary antibodies were used: MDM2 (sc-965, Santa Cruz Biotechnology), SEMA6A (ab72369, Abcam), SFRP1 (ab126613, Abcam), shugoshin 1 (ab21633, Abcam) and TTK (3255S, Cell Signaling). Beta-actin antibody (4970, Cell Signaling) was used as a loading control. For secondary antibodies, either goat anti-rabbit HRP (sc-2004, Santa Cruz Biotechnology) or goat anti-mouse HRP (sc-2005, Santa Cruz Biotechnology) were used. Signals were detected by using a Lumigen TMA-6 reagent (Lumigen Inc).

### Statistical analysis

Student *t-test* was applied to compare the significances between control and siRNA transfected counterparts. P value ≤0.05 is considered as significant.

## Results

### Analysis of microarray targets

HCC1954 is a Her2+ breast cancer cell line that displays approximately 10% CA in unsynchronized populations, significantly higher compared to MCF10A non-transformed cells [4,15,16]. In a parallel microarray assay (Lee and Saavedra, unpublished), we aimed to identify genes differentially expressed between HCC1954 cells silenced for E2F3 and cells expressing empty vector control (HCC1954/pLKO.1). For that purpose, we used the lentiviral pLKO.1-*puro* shRNA system to silence E2F3. The microarray analysis presented here compared the gene expression between HCC1954 cells and MCF10A cells and was carried out in HCC1954 cells expressing the empty lentiviral pLKO.1-*puro* vector. For consistency, MCF10A/pLKO.1 non-tumorigenic cells were used as comparison. We first selected the top 20% genes that were differentially distributed across the microarray samples and performed Metacore gene enrichment analysis. The selected targets fell into various categories, with genes involved in S phase regulation and DNA damage checkpoint control being the most highly represented (Table 2). Our initial screening generated 2135 genes under expressed in HCC1954 versus MCF10A cells. On the other hand, the microarray data identified 2635 genes upregulated in HCC1954 cells relative to MCF10A. Following the analysis for centrosome and cell cycle GO processes, we narrowed down our findings to genes with ≥1.5 fold higher expression in MCF10A vs HCC1954 cells and found 169 for cell cycle and 7 for centrosome with an overlap of 3 genes between the two GOs. The downstream GO analysis indicated that 421 genes with ≥1.5 higher expression in HCC1954 cells were involved in the cell cycle, 23 were linked to the centrosome and 21 genes pertained to both GOs (Table 3).

**Table 2:** Enrichment analysis report by process networks

**Number**	**Networks**	**HCC1954 vs MCF10A (P value)**	**Genes**
1	Cell cycle, S phase	6.7436787067153E-13	RFC4, TOP2 beta, Chk2, MCM3, SG2NA, NFBD1, Brca1, Cyclin B, Cyclin B2, CRM1, CHMP1A, ORC1L, MCM5, Nek2A, MCM4, PRIM2A, MCM10, ORC3L, DERPC, Histone H1.5, ChAF1 subunit B, ORC6L, ASK (Dbf4), POLA1, POLE2, FEN1, DNA ligase I, p21, RFC5, BUB1, Geminin, Sgo1, Rad51, DNA polymerase alpha/primase, PRIM1, HP1, Securin, CDK1 (p34), C-Nap1(CEP2), Ubiquitin, PCNA, CDC18L (CDC6), Cyclin A1, UHRF2, BRIP1, RFC3, POLD reg (p68), ATM, TOP2 alpha, POLE3-POLE4 complex, MCM6, POLA2, GADD45 alpha, RFC1, RFC2, Cyclin A, DNA polymerase sigma, PCTK1, TOP2, ZAK, Cdt1, Cyclin B1, POLE3 (YBL1), p53, RFC complex, Emi1, Nibrin, PKA-cat (cAMP-dependent), Rad21, RPA2, Histone H4, DCC1, Histone H1, CDC45L
2	DNA damage checkpoint	9.94791617812864E-08	RFC4, CIA/ASF1, p38alpha (MAPK14), Chk2, 14-3-3 epsilon, NFBD1, Brca1, Cyclin B, Cyclin B2, Cyclin D3, HUS1, CDC25C, ANAPC11, ERK1/2, Cyclin D, 14-3-3, Rad50, CDC25A, BTG2, p21, RFC5, Securin, CDK1 (p34), ANAPC7, Cyclin D2, Ubiquitin, PCNA, CDC23, Cyclin A1, MRE11, BRIP1, RFC3, MRN complex, FANCD2, RAD1, ATM, MDM2, GADD45 alpha, RFC1, Ku70, RFC2, Cyclin A, Cyclin D1, JNK(MAPK8-10), NF-kB, 14-3-3 eta, p38gamma (MAPK12), RUVBL2, p53, RFC complex, Bard1, USP1, Nibrin, p38 MAPK, Rad21
3	Cell cycle, mitosis	3.45421335237624E-07	Tubulin beta, Tubulin gamma, MIS12, HZwint-1, Cyclin B, Cyclin B2, ZW10, CAPZ beta, Nek2A, CDC25C, Tubulin alpha 1A, CENP-A, ANAPC11, MACF1, MAD2a, CENP-H, SPBC25, Importin (karyopherin)-alpha, Rod, RCC1, CAP-D2/D3, Kid, CDC25A, USP16, BUB1, MPP6, Dynamin-2, Aurora-A, Actin, CDC25, CDCA1, HP1, Securin, CDK1 (p34), SIL, ANAPC7, Ubiquitin, CDC23, PARP-2, Tubulin alpha, Dynein 1, cytoplasmic, heavy chain, Tctex-1, Survivin, Karyopherin alpha 2, MAPRE3(EB3), Dynamin-3, ANAPC10, DLC1 (Dynein LC8a), BUBR1, CAP-H/H2, NF45 (ILF2), Cyclin A, EML4, HEC, TTK, CAS-L, TOP2, MAD2L1BP, 14-3-3 eta, Dynamin, MCAK, Cyclin B1, PAFAH alpha (LIS1), RAE1, PARD6, HP1 gamma, Rad21, Zwilch, NSL1, Histone H1, Tubulin (in microtubules)
4	Cell cycle, G2-M	8.54322861724783E-07	TOP2 beta, p38alpha (MAPK14), Chk2, TCP1-theta, 14-3-3 epsilon, EGF, TCP1-delta, NFBD1, Brca1, Cyclin B, Cyclin B2, GRB2, HUS1, Nek2A, CDC25C, HIPK2, p38delta (MAPK13), EGFR, ANAPC11, NEDD8, MAD2a, ERK1/2, 14-3-3, Histone H1.5, Centrin-2, RCC1, Rad50, CAP-D2/D3, Kid, CDC25A, p21, BUB1, Rad51, CDK10, Aurora-A, CDC25, p90Rsk, Securin, CDK1 (p34), RINT-1, ANAPC7, Ubiquitin, FHIT, CDC23, Cyclin A1, Shc, CKS1, TRF1, FANCD2, ATM, AKT3, MRLC, Lamin B1, TCP1-epsilon, BUBR1, TOP2 alpha, CAP-H/H2, LATS2, Lamin B, IGF-1 receptor, GADD45 alpha, Cyclin A, CNAP1, PCTK1, TOP2, AKT(PKB), ZAK, 14-3-3 eta, Dynamin, BRRN1, p38gamma (MAPK12), Cyclin B1, p53, Emi1, p38 MAPK, PKA-cat (cAMP-dependent), MAT1, Histone H1
5	DNA damage, BER-NER repair	1.15885388728944E-06	RFC4, Chk2, OGG1, NEIL3, NFBD1, Brca1, PARP-1, HUS1, MBD4, NEIL1, ERCC6, Rad50, DNA polymerase beta, POLE2, XPF, FEN1, DNA ligase I, RFC5, TFIIH p52 subunit, TDG, TFIIH p34 subunit, PCNA, UNG1, PARP-2, MRE11, DDB2, RFC3, RAD23B, MRN complex, RAD1, POLD reg (p68), ATM, PARG, POLE3-POLE4 complex, RFC1, RFC2, Brca1/Bard1, PARP-3, POLE3 (YBL1), p53, RFC complex, Bard1, Nibrin, MBD1, XPD, RPA2, MAT1, TFB5
6	Cell adhesion, cell junctions	7.60204508182795E-06	Tubulin beta, Tiam1, VE-cadherin, BPAG2, N-cadherin, E-cadherin, ITGB1, 14-3-3 epsilon, Cingulin, MUPP1, Tcf(Lef), ZO-1, Fer, ERK1/2, Keratin 5, 14-3-3, Claudin-16, Beta-catenin, Nectin-2, BPAG1, DLG5(P-dlg), Connexin 46, Claudin-8, Desmoglein 3, Vimentin, Actin, Paxillin, MAGI-1(BAIAP1), GIT1, PI3K reg class IA (p85), JAM3, ATP1B1, Tubulin alpha, Desmocollin 3, R-cadherin, PKC, PLC-beta, Endothelin-1, CASK, Keratin 18, Desmoglein 2, Beta-fodrin, Claudin-7, Itch, PLC-gamma, Keratin 8/18, Claudin-4, SIP1 (ZFHX1B), Nectin-3, Desmoplakin, Keratin 8, ZO-3, PSD-95, Keratin 6A, PEZ, ERK2 (MAPK1), Caveolin-1, 14-3-3 eta, Claudin-3, PARD6, Connexin 31, Tubulin (in microtubules)
7	Cell cycle, core	0.000013064502255225	MCM3, p14ARF, Cyclin B, Cyclin B2, Cyclin D3, ZW10, ORC1L, MCM5, Nek2A, CDC25C, CENP-A, MCM4, MCM10, ORC3L, MAD2a, CENP-H, Cyclin D, Rod, Kid, CDC25A, ORC6L, ASK (Dbf4), FEN1, DNA ligase I, p21, BUB1, DNA polymerase alpha/primase, Aurora-A, Securin, CDK1 (p34), Cyclin E2, CDC18L (CDC6), Survivin, BUBR1, MCM6, Cyclin A, Cyclin D1, HEC, TOP2, Cdt1, Cyclin B1, p16INK4, Emi1, RPA2, Zwilch, MAT1, CDC45L
8	Cell adhesion, cadherins	0.0000405298162912831	RACK1, WNT4, VE-cadherin, BPAG2, N-cadherin, E-cadherin, ITGB1, PCDHA6, PCDHA4, VLDLR, Tcf(Lef), EGFR, Ski, ZO-1, Presenilin 1, Fer, DKK1, PCDHGC3, Casein kinase I epsilon, Beta-catenin, Nectin-2, H-cadherin, PTPR-mu, SSX2IP, BPAG1, DLG5(P-dlg), PCDHGB2, Desmoglein 3, Actin, CTNNAL1, Protocadherin 18, PCDHGA3, Shc, PI3K reg class IA (p85), PCDA7, Desmocollin 3, R-cadherin, MTSS1, Cadherin 10, FAT1, PKC, PCDHGA1, FHL2, PKC-alpha, Protocadherin gamma B1, Desmoglein 2, WNT, WNT10B, EVL, Nectin-3, LRP6, Axin2, Casein kinase I, PCDHGA12, Fyn, Vinexin, DAB1, PEZ, AKT(PKB), PDZK3, PCDHGB4, Axin, PTPR-zeta, Frizzled, Tubulin (in microtubules)
9	Proteolysis ubiquitin-proteasomal proteolysis	0.0000769830526795964	MDM4, HAUS7, Brca1, PSMB8(LMP7), KEAP1, PSMA4, Cullin 2, c-Cbl, UBCH7, UCHL3, SAE1/2, TGT, PSMB1, PSMC3, PSMC6, PSMD5, PSMA7, PSMC4, SENP2, SAE1, GRAIL, PSMD10 (Gankyrin), PSME1, DTX3, Ubiquitin, PSMD3, PSMD2, RAD23B, NF-X1, PSMB6, MUF1, PSME2, PSMD8, PSMD7, Syntaxin 5, Itch, Elongin C, MPDZ, NEDD4L, MDM2, DORFIN, PSMB3, UBC7, PSMD14, Brca1/Bard1, RING-box protein 1, PSMB7, PSMD12, PSMA3, PSMC5, BAG-1, PSMA5, HSP70, p47, Bard1, PSMA6, PSMA2, PSMD6, PSMC1, MMS2
10	Cytoskeleton spindle microtubules	0.00008172060696576	Tubulin beta, Tubulin gamma, KIF4A, HZwint-1, Cyclin B, HOOK1, Cyclin B2, ZW10, Nek2A, Tubulin alpha 1A, CENP-A, Tubulin delta, MAD2a, CENP-H, Importin (karyopherin)-alpha, Rod, RCC1, MKLP2, Kid, BUB1, Sororin, Aurora-A, Securin, CDK1 (p34), ANAPC7, Tubulin alpha, Dynein 1, cytoplasmic, heavy chain, Karyopherin alpha 2, 4.1N, DLC1 (Dynein LC8a), BUBR1, DORFIN, DEEPEST, MID1, EML4, HEC, TTK, MCAK, Cyclin B1, PAFAH alpha (LIS1), RAE1, Zwilch, Tubulin (in microtubules)

**Table 3:** Deregulated centrosome genes

**Gene symbol**	**Gene name**	**HCC1954/MCF10A**
PLK2	polo-like kinase 2	5.82
SGOL1	shugoshin-like 1 (S. pombe)	4.24
CETN2	centrin, EF-hand protein, 2	4.12
AURKA	aurora kinase A	4.01
BRCA1	breast cancer 1, early onset	3.77
CDK1	cyclin-dependent kinase 1	3.50
SON	SON DNA binding protein	3.09
NEK2	NIMA (never in mitosis gene a)-related kinase 2	2.89
RANBP1	RAN binding protein 1	2.82
CEP76	centrosomal protein 76 kDa	2.46
GADD45A	growth arrest and DNA-damage-inducible, alpha	2.40
TMEM67	transmembrane protein 67	2.40
TBCCD1	TBCC domain containing 1	2.36
SASS6	spindle assembly 6 homolog (C. elegans)	2.33
UXT	ubiquitously-expressed, prefoldin-like chaperone	2.27
XPO1	exportin 1 (CRM1 homolog, yeast)	2.13
HAUS2	HAUS augmin-like complex, subunit 2	2.13
CEP192	centrosomal protein 192 kDa	2.03
HAUS7	HAUS augmin-like complex, subunit 7	2.03
SPICE1	spindle and centriole associated protein 1	2.01
CTNNB1	catenin (cadherin-associated protein), beta 1, 88 kDa	1.88
HAUS5	HAUS augmin-like complex, subunit 5	1.65
PAFAH1B1	platelet-activating factor acetylhydrolase 1b, regulatory subunit 1 (45 kDa)	1.50
NDEL1	nudE nuclear distribution E homolog (A. nidulans)-like 1	−1.50
ARHGEF10	Rho guanine nucleotide exchange factor (GEF) 10	−1.76
PKD2	polycystic kidney disease 2 (autosomal dominant)	−1.80
PCM1	pericentriolar material 1	−1.82
CEP250	centrosomal protein 250 kDa	−1.89
PLXNA2	plexin A2	−3.92
SEMA6A	sema domain, transmembrane domain (TM), and cytoplasmic domain, (semaphorin) 6A	−6.99

### Validation of microarray targets

**Figure 1:**
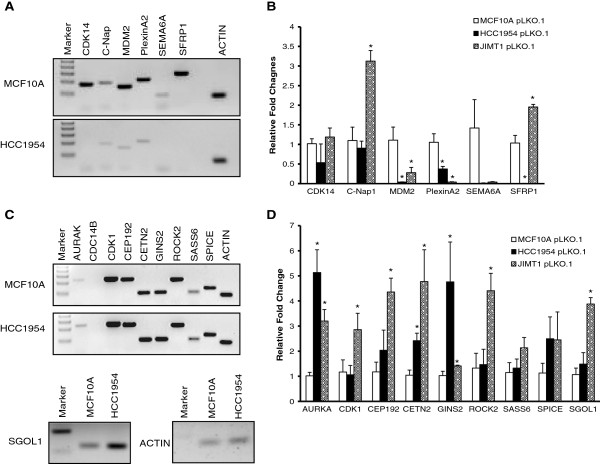
**Differential expression of cell cycle regulators and centrosomal genes between MCF10A cells and Her2+ breast cancer cells.** Semi-quantitative PCR **(A)** and real time PCR analysis **(B)** of genes downregulated in Her2+ breast cancer cells compared to MCF10A cells. Semi-quantitative PCR **(C)** and real time PCR analysis **(D)** of genes upregulated in Her2+ breast cancer cells compared to MCF10A cells.

Based on fold changes and our interests, we selected genes that were upregulated (AURKA, CDC14B, CDK1, CEP192, CETN2, GINS2, ROCK2, SASS6, SPICE, TTK and SGOL1) as well as downregulated (CDK14, C-Nap1, MDM2, PlexinA2, SEMA6A, and SFRP1) in HCC1954 cells compared to non-tumorigenic MCF10A cell line. In addition, JIMT-1 cells, a second Her2+ cell line with high CA [4,16], were included in this analysis to investigate the similarity of molecular patterns between two different Her2+ cell lines (Table 4). Semi-quantitative PCR analysis validated the differential expression for most genes downregulated in HCC1954 cells (Figure 1A) and for some genes upregulated in this cell line (Figure 1C). Consistent with this finding, similar trends were found by real-time PCR analysis (Figure 1B,D). The results show that, compared to MCF10A control, MDM2 and PlexinA2 were significantly downregulated in JIMT-1 and in HCC1954 cells. On the other hand, C-Nap1 and SFRP1 RNA levels were significantly increased in JIMT-1 cells compared to MCF10A cells. Among the upregulated genes in HCC1954 cells, AURKA, CETN2 and GINS2 RNA levels were significantly increased compared to MCF10A and all of the genes investigated, with the exception of SAS6 and SPICE, were more highly upregulated in JIMT-1 cells than in HCC1954 cells. These data validated our microarray analysis and confirmed differences of molecular signatures between two Her2+ breast cancer cell lines.

**Table 4:** Differential expression of selected cell cycle and centrosome genes

**Accession number**	**Genes down regulated in breast cancer cells**	**Metacore fold change**	**Fold changes, real-time PCR**	
			**HCC1954**	**JIMT-1**
[GenBank: NM_012395]	CDK14	14.26	0.53 ± 0.48	1.19 ± 0.23
[GenBank:NM_014865.3]	C-Nap1	1.89	0.90 ± 0.18	3.13 ± 0.27
[GenBank:NM_002392]	MDM2	3.18	0.04 ± 0.01	0.28 ± 0.13
[GenBank:NM_025179]	Plexin A2	3.92	0.37 ± 0.07	0.04 ± 0.01
[GenBank:NM_020796]	Semaphorin 6A	6.99	0.01 ± 0.01	0.04 ± 0.01
[GenBank:NM_003012]	SFRP1	38.04	0.00 ± 0.00	1.96 ± 0.07
	**Genes up regulated in breast cancer cells**			
[GenBank:NM_003318]	TTK	4.62	2.10 ± 1.11	N/A
[GenBank:NM_198433]	Aurora-A kinase	4.01	5.14 ± 0.91	3.2 ± 0.46
[GenBank:NM_001786]	CDK1	3.5	1.06 ± 0.37	2.85 ± 0.65
[GenBank:NM_032142]	CEP192	2.03	2.03 ± 0.81	4.36 ± 0.55
[GenBank:NM_004344]	Centrin-2	4.12	2.42 ± 0.30	4.77 ± 1.27
[GenBank:NM_016095]	GINS2	11.49	4.76 ± 1.59	1.41 ± 0.04
[GenBank:NM_004850]	ROCK2	<1.5	1.46 ± 0.61	4.41 ± 0.70
[GenBank:NM_194292]	SASS6	2.33	1.33 ± 0.36	2.13 ± 0.41
[GenBank:NM_144718]	SPICE1	2.01	2.50 ± 0.87	2.45 ± 1.11
[GenBank:NM_001012410]	SGOL1	4.24	1.49 ± 0.46	3.88 ± 0.26

### Transient knockdown of SEMA6A or MDM2 and centrosome amplification in MCF10A cells

**Figure 2:**
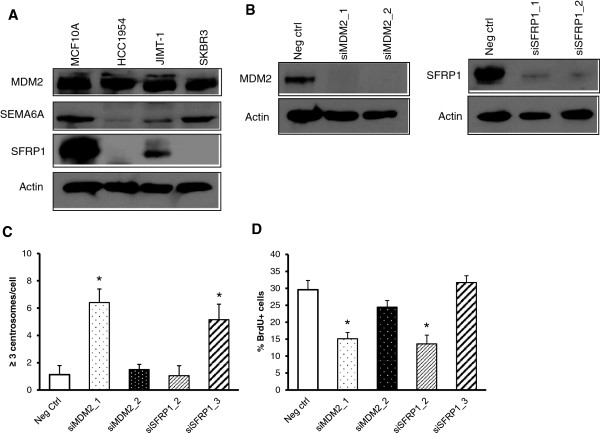
**MDM2 and SFRP1 in centrosome amplification. (A)** Basal levels of MDM2, semaphorin 6A and SFRP1 are higher in unsynchronized populations of MCF10A cells compared to Her2+ breast cancer cell lines. **(B)** MCF10A cells were transfected with siRNA duplexes against MDM2 or SFRP1. Western blots indicate the transient knockdown of MDM2 and SFRP1. Quantifications of centrosome amplification by pericentrin immunofluorescence **(C)** and of BrdU incorporation by fluorescence microscopy **(D)**.

After the real-time PCR validation of the microarray targets, we chose to further pursue three genes; MDM2, SEMA6A and SFRP1 that were downregulated in HCC1954 cells, based on the relevance and novelty of their function in centrosome duplication. First, we investigated protein expression by Western blot (Figure 2A). Both SEMA6A and SFRP1 protein levels were highly decreased in three Her2+ cell lines, HCC1954, JIMT-1 and SKBR3 compared to non-transformed normal epithelial MCF10A cells. However, unlike real-time PCR data, MDM2 protein levels across the three Her2+ cell lines were comparable to those of MCF10A cells. Since two of these proteins were less abundant in breast cancer cells, we speculated that at high levels, these molecules would represent suppressors of CA. To test this possibility, we proceeded to knock these genes down in MCF10A cells using siRNAs to test whether their downregulation induces CA. As illustrated in Figure 2B, we were able to effectively knockdown both MDM2 and SFRP1 with two independent sequences targeting different regions of the genes. However, we did not achieve knockdown of SEMA6A using the same strategy; thus, we eliminated this gene from further experiments. Even though both siRNA sequences significantly decreased MDM2 and SFRP1 protein levels in MCF10A cells, only one duplex for each gene (MDM2_1, SFRP1_3) induced CA (Figure 2). Similar results were obtained for the BrdU incorporation assay, since only one sequence (MDM2_1, SFRP1_2, respectively) decreased the extent of BrdU incorporation.

### Transient knockdown of SGOL1 or TTK decreases centrosome amplification in Her2+ cells

**Figure 3:**
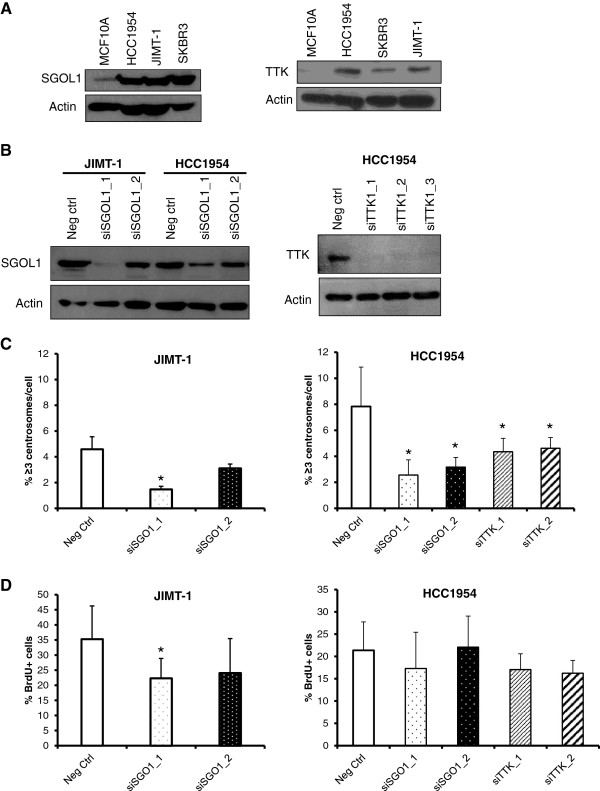
**Shugoshin 1 and TTK maintain centrosome amplification in Her2+ breast cancer cells. (A)** Basal levels of SGOL1 and TTK are higher in unsynchronized populations of Her2+ breast cancer cell lines compared to MCF10A cells. JIMT-1 cells were transfected with siRNA duplexes against SGOL1 and HCC1954 cells were transfected with siRNA against SGOL1 or TTK. **(B)** Western blots indicating the transient knockdown of SGOL1 in JIMT-1 and HCC1954 cells (left) and TTK in HCC1954 cells (right). Quantifications of fluorescent microscopy for centrosome amplification by pericentrin staining **(C)** and of BrdU incorporation **(D)**.

From the subset of genes that were upregulated in HCC1954 cells compared to MCF10A cells, we chose shugoshin 1 (SGOL1) and TTK, based on their relevance and novelty, since the centrosome roles of these genes are not fully understood, especially in cancer models. We predicted that their gene products are required for CA and proceeded to silence them in order to address whether their silencing would diminish percentages of CA in Her2+ breast cancer cells. Western blot analysis shown in Figure 3A confirmed that protein levels of SGOL1 and TTK were much higher in unsynchronized populations of Her2+ cell lines compared to MCF10A cells. Next, we transiently knocked down both proteins by using at least two independent siRNA duplexes, achieving total silencing with SGOL1_1 and partial downregulation with SGOL1_2 (Figure 3B). Knockdown of SGOL1 with SGOL1_1 decreased the percentages of CA in both HCC1954 and JIMT-1 cell lines compared to scrambled negative control and the same clone reduced percentages of BrdU + cells in JIMT-1 cells (Figure 3C,D). Knockdown of TTK with two independent siRNA sequences reduced the percentages of CA in HCC1954 cells (Figure 3C) and modestly affected BrdU incorporation (Figure 3D). These data suggest that reduced CA detected in HCC1954 and JIMT-1 cells by transient knockdown of SGOL1 may derive from the reduced DNA replication. However, the reduction in CA observed after transient knockdown of TTK is due to a mechanism apart from changes in DNA replication.

### Association of MDM2, SFRP1, and TTK expression with the outcome of breast cancer patients

**Figure 4:**
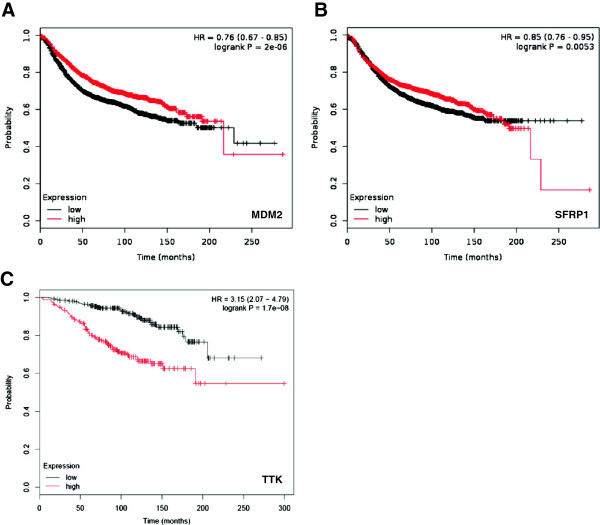
**Correlations between the levels of MDM2, SFRP1 or TTK and survival of breast cancer patients. (A)** Kaplan-Meier curve for MDM2 expression and relapse-free survival of breast cancer patients of all subtypes. **(B)** Kaplan-Meier curve for SFRP1 expression and relapse-free survival of breast cancer patients of all subtypes. **(C)** Kaplan-Meier curve for TTK expression and overall survival of luminal A breast cancer patients.

To explore the clinical relevance of the target genes, we used the http://kmplot.com/analysis/ resource that provides an online survival analysis tool to rapidly assess the effect of 22,277 genes on breast cancer prognosis using microarray data of 1809 patients [20]. We found that MDM2 and SFRP1 transcripts were positively correlated with relapse free survival for all breast cancer subtypes and the results were statistically significant (Figure 4A,B). In the case of TTK, RNA level was negatively correlated with overall survival of luminal A breast cancer patients, showing statistical significance (Figure 4C). However, no public survival data were available for SGOL1.

## Discussion

Centrosome amplification (CA) is a precursor of chromosome instability and polyploidy and may facilitate the acquisition and maintenance of several malignant phenotypes in breast tumors. Previously, we screened breast cancer cell lines of various subtypes to find an accurate model for CA studies and found three Her2+ cell lines; HCC1954, JIMT-1 and SKBR3 that displayed significant CA compared to MCF10A non-tumorigenic epithelial cells [4,16]. To seek out new targets differentially expressed in these cell lines, thereby potentially modulating the extent of CA, we performed a microarray based on Affymetrix platform using MCF10A cells and HCC1954 cells as a starting point and explored several selected targets by real time PCR and Western blots.

Amongst the differentially expressed genes, we identified downregulation of secreted frizzled-related protein-1 (SFRP-1) in three Her2+ breast cancer cell lines. SFRPs are inhibitory WNT family members that block the WNT signaling pathway and the competition between Frizzled and SFRP1 for binding to WNT regulates the pathway activation [21]. There is a plethora of evidence of loss of SFRP1 expression in many cancers including breast cancer [22-25], suggesting that this gene acts as a mammary tumor suppressor. SFRP1 is expressed in the normal breast epithelium and its expression is lost in more than 80% of invasive breast carcinomas [26]. In a mouse model, 10 week old nulliparous SFRP1-/- animals showed inappropriate mammary gland development relative to controls, evidenced by extensive branching with clear lobulo-alveolar development, along with a significantly higher density of ducts with distinct alveoli present throughout the mammary gland [27]. More recently, Matsuda *et al* showed that ectopic expression of SFRP1 in MDA-MB-231 triple negative breast cancer cells blocks canonical WNT signaling and decreases their migration potential and cell proliferation in a xenograft model along with dramatically impairing lung metastases [28]. Another relevant study showed that triple negative breast cancer cells treated with HDAC inhibitor romidepsin and methyltransferase inhibitor decitabine induced reexpression of SFRP1, along with synergistic inhibition of cell growth and induction of apoptosis [29]. To date, there have been no reports about the function of SFRP1 in regards to CA. In this study, we show that in normal epithelial MCF10A cells, SFRP1 knockdown with siRNA sequences did not consistently induce CA.

MDM2, mouse double minute 2 homolog, first identified as an inhibitor of p53 transcriptional activation [30], is an E3 ubiquitin ligase that targets p53 degradation, thereby keeping p53 levels and activity low in unstressed cells [31]. The molecular significance of MDM2 stems from its ability to interact with more than 100 molecules [32]. For instance, activation of E2F1 is stimulated by its binding to MDM2 [33]. Amplification or altered expression of MDM protein has been found in many tumors [34-36]. In terms of its breast cancer-promoting properties, Smad3/4 transcription factors activated by TGF-β1 bind to the promoter region of MDM2 to increase its protein expression [37]. As a result, upon TGF-β1 treatment, the murine mammary epithelial cells NMuMg undergo the epithelial to mesenchymal transition and are able to migrate. Also, MDM2 overexpression in MCF-7 (estrogen receptor positive, Her2-negative) and MDA-MB-231 (triple-negative) cell lines promotes invasion and metastasis in invasive ductal breast carcinoma by upregulating matrix metalloproteinase-9 [38]. There have been a handful of reports about overexpression of MDM2 and CA, but never one that addresses the effects of its downregulation and CA. For example MDM2 overexpression promotes CA in tumors that retain wild-type p53 [39]. However, later studies showed that the functions of MDM2 in inducing genomic instability and transformation could also be p53-independent [40-43]. Our validated microarray revealed highly decreased mRNA levels of MDM2 in Her2+ cells compared to MCF10A but MDM2 protein level was not diminished. Similar to SFRP1, knockdown of MDM2 with siRNA did not consistently induce CA. In this report, we found that lower MDM2 mRNA expression negatively correlates with disease-free survival. In summary, the mechanisms responsible for the acquisition of SFRP1 or MDM2-mediated CA need to be further investigated and one interesting aspect is whether SFRP1 or MDM2 overexpression in Her2+ cells decreases CA.

From the 2635 genes overexpressed in HCC1954 Her2+ breast cancer cells versus non-tumorigenic control and following downstream analyses, shugoshin 1 and TTK piqued our interest. These genes are of interest due to potential diverse functions associated with chromosome segregation, spindle formation and centrosome cycle. The impact of these genes on CA is also unclear and controversial. We did not study Aurora A kinase, the top centrosome upregulated gene, since Aurora A overexpression is known to promote CA [44,45]. Likewise, Nek2 is another kinase we did not address, since our previous publications indicate it maintains CA and CIN in Her2+ breast cancer cells [15,16] and in MCF10A cells expressing H-Ras^G12V^ or H-Ras^G12V^ and c-Myc [46]. Shugoshin proteins (SGOL1 and SGOL2) prevent the cleavage of cohesion complexes localized around sister chromatids by separase from S phase to metaphase. In vertebrates, this function is probably achieved via the interaction between SGOL1 and the PP2A phosphatases that counteract the phosphorylation of cohesion subunits [47,48]. SGOL1 interacts with the spindle assembly checkpoint (SAC) kinase Bub1 and this interaction is required for the centromere localization of shugoshin [49-52]. Centromeric localization of SGOL1 is also promoted by the interaction with Aurora B kinase of the chromosomal passenger complex (CPC) [52]. Finally, because of phosphorylation by Nek2, SGOL1 integrates the centromeric cohesion and spindle attachment at the kinetochore [53]. Although not very abundant, the current literature clearly points out that shugoshin can accomplish multiple functions, such as chromatid and centriole cohesion or spindle assembly. Therefore, SGOL1 is crucial for chromosome segregation and mitotic progression and its deregulation can have detrimental effects including centrosome amplification, chromosome instability, and aneuploidy. Depletion of SGOL1 precludes viability as demonstrated by *Sgol 1* knockout mouse studies [50]. Overexpression of SGOL1 was detected in the sera of breast cancer patients [54] and has been associated with additional human cancers, including gastric, colorectal, AML and NSCLC [48,55-57]. In stark contrast with these clinical correlations, another study in colorectal cancer found low levels of SGOL1 in tumor samples while in cell culture SGOL1 knockdown induced CA, CIN and mitotic catastrophe [58]. A genome-wide RNAi array performed in oral squamous cancer cells detected SGOL1 as one of the molecules implicated in centrosome clustering [59]. This is a phenomenon that typically occurs in cells with amplified centrosomes and leads to the formation of pseudopolar spindles and bipolar division, as opposed to mitotic multipolar spindles that cause cell death [10]. Various functional assays demonstrated that transient silencing of SGOL1 resulted in mitotic arrest, multipolar spindles, reduced spindle tension, and cell death [59]. This seminal work showing that depletion of SGOL1 inhibits centrosome clustering is consistent with our data confirming a linear correlation between the extent of SGOL1 knockdown and the decrease of centrosome amplification, as measured by pericentrin labeling, and also a correlation with decreased DNA synthesis. Our findings presented here represent the first evidence of the biological relevance of SGOL1 in a breast cancer cell model and the first indication that its overexpression promotes CA. In conclusion, the versatile SGOL1 protein may coordinate critical interactions involving the spindles and chromosome biology. As a result, deregulated SGOL1 localization and function in breast cancer cells can significantly contribute to the acquisition of CA, centrosome clustering, chromosome missegregation and instability and aneuploidy. Based on this evidence, SGOL1 is an attractive target for blocking the progression of genomically unstable breast tumors that display CA. However, the molecular biology underlying the centrosome and centromere functions of SGOL1 awaits more studies.

TTK, also known as monopolar spindle 1, is a dual specificity kinase with well characterized roles in the spindle assembly checkpoint [60]. In addition to its function during mitosis, TTK has also shown a role in centriole duplication and assembly [61,62]. Proper levels and regulation of TTK are partially responsible for ensuring accurate centriole duplication and assembly during the cell cycle [63]. One mechanism which prevents TTK accumulation from resulting centriole re-duplication is through control of TTK levels via the MPS1 degradation signal [62]. Although TTK may not be required for normal centriole duplication, several studies support the notion that overexpressed TTK results in centriole re-duplication, which could lead to CA. In breast cancer, increased TTK mRNA levels have been noted across many cell lines, specifically Her2+ and triple negative subtypes and in tumor samples collected from patients with advanced disease [64-66]. These correlations suggest an association between TTK levels and cancer behavior (i.e. cell proliferation, tumor aggressiveness). Thus, TTK presents a potential biomarker to predict patient prognosis in breast cancer or as a useful drug target for a subset of breast cancers. Previous studies have revealed how modifying TTK expression levels affects cell viability, mitosis and tumor growth *in vivo* but none have addressed TTK’s functional roles involved with CA in breast cancer [64,65]. In this report, we show for the first time that attenuating TTK levels can decrease the percentages of CA observed in a subset of Her2+ breast cancer cells without affecting the integrity of DNA synthesis. Further dissection of the mechanism for how TTK can drive CA and genomic instability in specific breast cancer subtypes will be a key to understanding the correlations between high TTK levels and more aggressive/drug resistant breast tumors.

## Abbreviations

CA: Centrosome amplification; CIN: Chromosome instability; ER: Estrogen receptor; PR: Progesterone receptor; DCIS: Ductal carcinoma *in situ*; MDM2: Mouse double minute 2 homolog; SFRP1: Secreted frizzled-related protein 1; SGOL1: Shugoshin-like 1; BrdU: 5-bromo-2-deoxyuridine; DAPI: 4′,6-diamidino-2-phenylindole; GO: Gene ontology; AML: Acute myeloid leukemia; NSCLC: Non-small cell lung cancer.

## Competing interests

The authors declare that they have no competing interests.

## Authors’ contribution

ML: Performed semi-quantitative and real-time PCR, siRNA knockdown of SFRP1 and MDM2, CA and BrdU assays and wrote part of manuscript. MM: Analyzed normalized microarray data, performed GO analysis, siRNA knockdown of SGOL1, CA and BrdU assays and wrote part of manuscript. JLK: Performed TTK real-time PCR, siRNA knockdown of TTK, CA and BrdU assays and wrote part of the manuscript. HIS: Designed the project and edited the manuscript. All authors read and approved the final manuscript.
